# Assessment of the intensity of state-trait anxiety of children with cancer

**DOI:** 10.1192/j.eurpsy.2024.465

**Published:** 2024-08-27

**Authors:** G. Kyritsi, A. Zartaloudi, H. Kyritsi, E. Dousis, E. Evangelou, C. Dafogianni, M. Polikandrioti, E. Vlachou, I. Koutelekos

**Affiliations:** ^1^Greek Health System; ^2^University of West Attica, Athens, Greece

## Abstract

**Introduction:**

Children with cancer face many difficulties on a daily basis which place them at increased risk of developing anxiety and discomfort.

**Objectives:**

To assess the intensity of state-trait anxiety in children with cancer.

**Methods:**

The sample of the study consisted of 100 children from Greek Children’s Hospital, aged 8-16 years, of which 56 had cancer, representing the study group while the control-group was 44 in an outpatient clinic with endocrinological problems. Data were collected by the completion of the questionnaire “State-Trait Anxiety Inventory for children” by Ch. Spielberger. Statistical package S.P.S.S. was used for statistical analysis. 22 and the statistical test, t-test and anova. The significance level was set at p <0.05.

**Results:**

Of the total sample, sarcoma 38%, brain Ca 14%, 48% endocrine problem, and the largest percentage (57%) were aged 8-10 years. Children with cancer in 44.6% were under treatment and 55.4% in remission or recovery. Body image change was experienced by the 44%. The mean value of the state anxiety was 30.3±5.4 and trait was 35.3±6.9. Children with cancer experienced lower levels of state anxiety compared to control group, p =0.049, and did not differed in terms of trait anxiety, p=0.060. In the total sample, girls experienced trait anxiety of the highest intensity, p=0.018 and children aged 14-16, p=0.020. No statistically significant differences were found in relation to the type of cancer in both state and trait anxiety, p=0.096 and p=0.424, in relation to the phase of the disease and the change of body image, p>0.05. Children whose fathers were of higher education experienced less anxiety and differed significantly from those of primary and secondary education, p=0.036 and p=0.021, respectively. Comparison between control group and study group in relation to gender, showed that girls with cancer experienced trait anxiety of higher intensity, p=0.029 but children between 14-16 years from the control group experienced trait anxiety of higher intensity, p=0.030.

**Conclusions:**

Children of both groups experienced mild to moderate anxiety and its intensity was related to socio-demographic factors of the children and their parents.

**Disclosure of Interest:**

None Declared

